# A targeted mass spectrometric assay for quantitative detection of fibrinogen in plasma

**DOI:** 10.1016/j.rpth.2026.106974

**Published:** 2026-09-18

**Authors:** Sandip Chavan, Ramesh Bokka, Shilpa Venkataraman, Anikha Bellad, Julie I. Tange, Rachel R. Leger, Rajiv K. Pruthi, Dong Chen, Jansen N. Seheult, Akhilesh Pandey

**Affiliations:** 1 Department of Laboratory Medicine and Pathology, Mayo Clinic, Rochester, Minnesota, USA; 2 Manipal Academy of Higher Education, Manipal, Karnataka, India; 3 Institute of Bioinformatics, International Technology Park, Bangalore, Karnataka, India; 4 Center for Individualized Medicine, Mayo Clinic, Rochester, Minnesota, USA

**Keywords:** blood coagulation, fibrinogen, selected reaction monitoring, tandem mass spectrometry, thrombosis

## Abstract

**Background:**

Fibrinogen is a key glycoprotein that plays a central role in clot formation, structure, and stability. Both quantitative fibrinogen deficiency and qualitative abnormalities can lead to bleeding or thrombotic disorders. Accurate quantification of fibrinogen antigen is essential for clinical evaluation; however, current immunoassays and clot-based surrogate methods are prone to interference and may lack traceability to international standards.

**Objectives:**

To develop a targeted mass spectrometry-based assay for accurate quantification of fibrinogen antigen in plasma and to compare its performance with established functional and immunoturbidimetric assays.

**Methods:**

We developed a targeted liquid chromatography-tandem mass spectrometry-based assay using isotope-labeled peptides that accurately measures fibrinogen antigen concentration with high precision (coefficient of variation < 20%) across a broad linear dynamic range of 10.2 to 1307.5 mg/dL, enabling accurate detection of hypofibrinogenemia and hyperfibrinogenemia. Assay performance was evaluated in healthy donors (*n* = 47) and clinical specimens (*n* = 31).

**Results:**

Fibrinogen antigen levels in normal donors followed a normal distribution (mean ± SD, 315 ± 42 mg/dL). The targeted assay, employing selected reaction monitoring, correlated strongly with immunoturbidimetry (Pearson’s correlation coefficient [r] = .943) and the Clauss and prothrombin time-derived fibrinogen assays (r = .91 and r = .93, respectively). Stratification by clinical category confirmed elevated fibrinogen levels in thrombosis-related disorders, intermediate levels in controls, and low levels in liver disease and critical illness.

**Conclusion:**

The liquid chromatography-tandem mass spectrometry-based assay provides precise, accurate quantification of fibrinogen across a wide clinical range and aligns closely with existing methods while offering analytical specificity. With further clinical validation, it could be used as a diagnostic clinical test.

## Essentials

• Conventional fibrinogen antigen assays are often limited by analytical interferences.
• A novel targeted mass spectrometry assay was developed and validated using isotope-labeled peptides.
• The assay demonstrated precise quantification across a wide dynamic range.
• Targeted LC-MS assay permits highly specific quantification of fibrinogen even in the presence of direct thrombin inhibitors.

## 1 Introduction

Fibrinogen is a complex glycoprotein produced and secreted by the liver that plays a key role in clot formation, structure, and stability [1]. It is a multimeric protein composed of 2 copies each of α, β, and γ chains, organized in a dimeric fashion (*Aα*,*Bβ*,*γ*)_2_ [[2], [3], [4]]. During hemostasis, thrombin cleaves short amino-terminal peptides from the α and β chains (fibrinopeptides A and B), exposing fibrin polymerization sites. This fibrin meshwork is the end product of the clotting machinery and, together with platelets, a key component of the blood clot (Figure 1). The fibrin polymer meshwork is further reinforced by isopeptide bonds between the γ chains of adjacent fibrin molecules and activated factor (F)XIII in circulation, which stabilizes the clot until the fibrinolytic pathway is activated. This pathway is regulated by complex mechanisms, and variations in the composition, concentration, and structural integrity (function) of fibrinogen, either congenital or acquired, can lead to defects in the clotting system [5]. These congenital or acquired fibrinogen disorders can lead to both bleeding and thrombotic manifestations [6,7].

**Figure 1:**
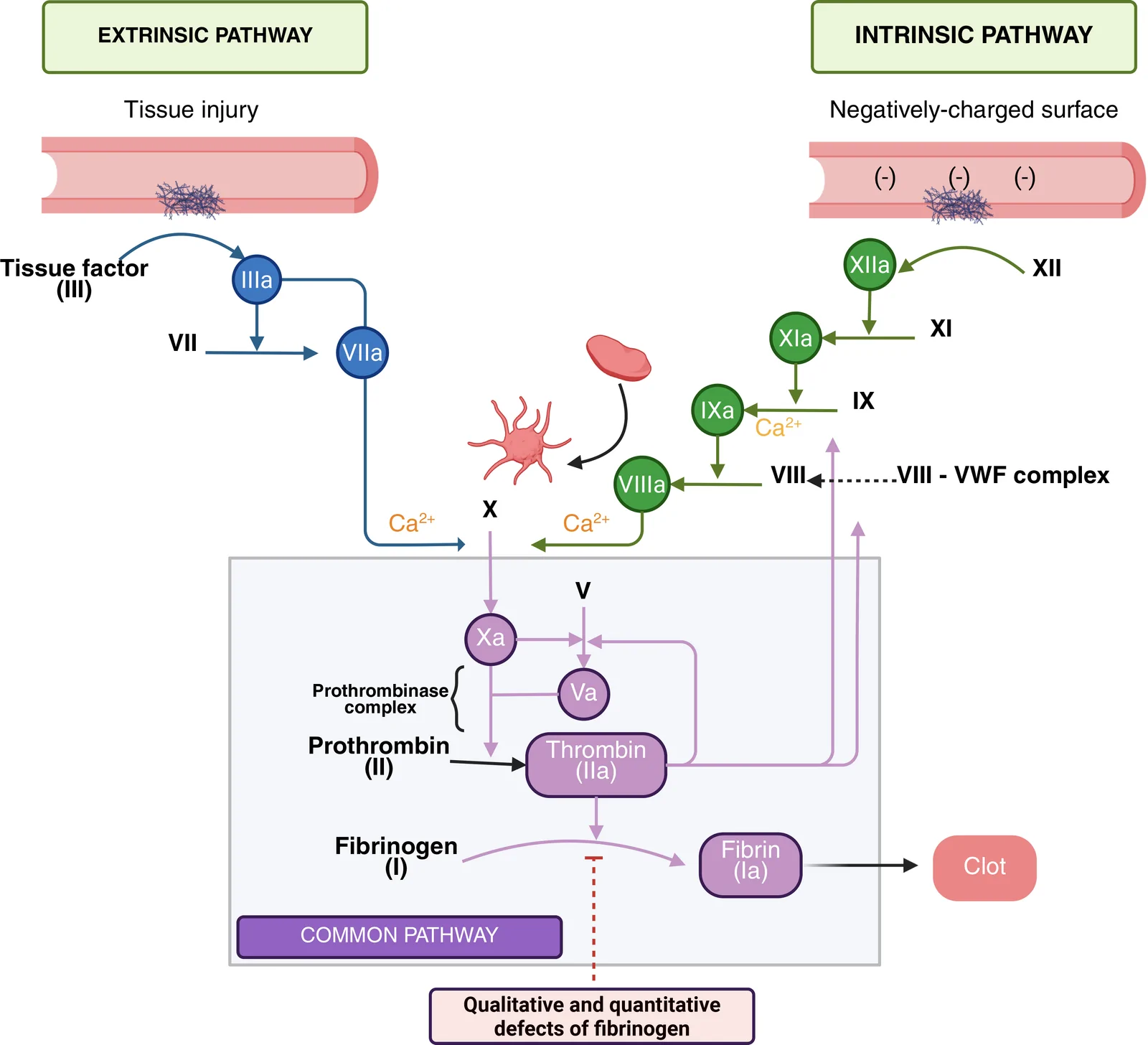
Schematic representation of the coagulation cascade. Tissue factors (tissue factor and factor [F]III) are activated upon tissue injury, which in turn initiates the extrinsic pathway. Negatively charged surfaces activate FXII in the intrinsic pathway. The prothrombinase complex (Xa-Va), formed upon convergence at FX, converts prothrombin to thrombin. Thrombin then cleaves fibrinogen into fibrin. Insoluble fibrin, cross-linked to form a mesh-like network, is produced from soluble fibrinogen. The coagulation cascade culminates in cross-linked fibrin. Calcium ions (Ca^2+^) and cofactors, including the FVIII-von Willebrand factor (VWF) complex, play critical regulatory roles.

Clinical laboratory investigation of fibrinogen disorders requires measuring functional fibrinogen based on fibrin clot formation kinetics and fibrinogen concentration [8]. The Clauss kinetic method is the most widely used laboratory technique for measuring clottable fibrinogen levels [9,10]. However, fibrinogen levels measured by the Clauss assay can be falsely decreased in the presence of anticoagulant drugs, such as direct thrombin inhibitors (DTIs), and substances that interfere with fibrin polymerization, such as fibrin degradation products (FDPs) and monoclonal proteins. Quantitative fibrinogen methods include immunological assays, which measure the total protein concentration of fibrinogen molecules regardless of whether they are functional [6,9], and the prothrombin time-derived fibrinogen (PT-Fib) curve on automated coagulation analyzers, which correlates more strongly with fibrinogen antigen concentration. Diagnosis of a qualitative fibrinogen defect requires comparison of Clauss fibrinogen with a measure of fibrinogen antigen concentration. Although the PT-Fib method is more widely available than immunoassay methods, it is subject to multiple interferences, including elevated FDPs and anticoagulant drugs, eg, DTIs. PT-Fib is therefore not recommended as an initial screening test since it can give misleading results in patients. In addition, any of the above methods that require spectrophotometric detection, eg, an optical coagulometer used for Clauss fibrinogen, are susceptible to false measurements in the presence of optical interfering substances, such as hemolysis, icterus, and lipemia.

There is a need for more accurate and precise analytical methods to quantify fibrinogen antigen levels, especially in the presence of interfering substances. An improved reference method is also needed to assign certified reference materials and improve metrological traceability. In this study, we developed a liquid chromatography-selected reaction monitoring (LC-SRM) method using stable isotope-labeled peptides to quantify fibrinogen α- and γ-chain antigens in patient and normal donor plasma samples. We further evaluated its performance by comparing the method with established functional and antigenic fibrinogen assays, including the Clauss assay, PT-Fib method, and immunological fibrinogen measurements.

## 2 Methods

### 2.1 Reagents and standards

Acetonitrile, formic acid, methanol, and water (liquid chromatography-mass spectrometry [LC-MS] grade) were purchased from Thermo Fisher Scientific. The World Health Organization Third International Standard for Fibrinogen Plasma (09/264) was obtained from the National Institute for Biological Standards and Control. CRYOcheck Normal Reference Plasma (#CCNRP-10) and CRYOcheck Abnormal 1 Reference Control (#ARP1-05) were purchased from Precision BioLogic. Owren-Koller buffer was purchased from BIOLABO. Iodoacetamide and ammonium bicarbonate (ABC) were purchased from Sigma-Aldrich. The Bond-Breaker TCEP Solution was obtained from Thermo Fisher Scientific. Peptides GSESGIFTNTK, ESSSHHPGIAEFPSR, TVIGPDGHK, and QFTSSTSYNR were synthesized for the fibrinogen α chain, while LDGSVDFK, VELEDWNGR, QSGLYFIKPLK, and YEASILTHDSSIR were synthesized for the γ chain. These peptides were produced using standard 9-fluorenylmethoxycarbonyl (FMOC) chemistry on a MultiPep RSi peptide synthesizer (CEM Corporation), as described previously [11]. Amino acid analysis was used to assess the salt content and purity (>95%) of all synthetic peptides (Supplementary Table S2).

### 2.2 Collection of clinical specimens and processing

Blood samples from individual patients and healthy donors were collected in 10 mL citrate tubes. For clinical samples, demographic information and the clinical indication for testing were abstracted from medical records; concurrent coagulation test results (eg, Clauss fibrinogen, prothrombin time [PT]/international normalized ratio, activated partial thromboplastin time [aPTT], D-dimer, and soluble fibrin monomer complex), along with the specimen collection date, were recorded (Table 1 and Supplementary Table S6). Tubes were inverted 10 times and kept on crushed ice for 30 minutes. The blood was then centrifuged at 1500 × *g* and 4 °C for 10 minutes. The separated plasma was stored in 2 mL aliquots at −80 °C for further experiments. The reference standards, calibrators, and samples were processed and digested similarly in a semiautomated manner using a 96-well plate and an automated liquid handler for C_18_ clean-up of samples. All samples were diluted 10-fold, and 10 μL of the diluted sample was reconstituted in lysis buffer (90 μL) composed of 8 M urea in 100 mM ABC and 5 mM Bond-Breaker TCEP Solution. All samples were spiked with 800 fmol (200 fmol/injection) of synthetic peptide standard mix, and the mixture was then incubated at 37 °C for 30 minutes. Samples were allowed to cool before being subjected to alkylation with iodoacetamide to a final concentration of 20 mM for 20 minutes at room temperature in the dark. The samples were then diluted with 100 mM ABC, followed by the addition of trypsin (tosyl phenylalanyl chloromethyl ketone [TPCK]-treated; Worthington Biochemical) at a 1:10 (w/w) enzyme-to-substrate ratio. The samples were then incubated at 37 °C overnight. The digested samples were subjected to C_18_ clean-up using the AssayMAP Bravo automated liquid handler (Agilent Technologies). The samples were dried using an SPE Dry system (Biotage) at 37 °C.

**Table 1:** Clinical diagnosis and demographic information of the tested samples.

ID	Sex	Age (y) at collection	Clinical history	Clauss result (mg/dL)	PT (s)	PT mix (s)	APTT (s)	aPTT mix (s)	TT (s)	RT (s)	PT-Fib result (mg/dL)	Immuno-turbidity result (mg/dL)	D-dimer (ng/mL)
1	F	29	No relevant history, normal coagulation studies	357	9.6		31		21.5		287	349	292
2	M	38	No relevant clinical history; elevated D-dimer and fibrinogen	556	9.8		29		18.9		666	580	3297
3	M	30	No relevant history, normal coagulation studies	254	12.7		32		23.1		271	267	<220
4	M	44	Autoimmune/inflammatory disease, elevated D-dimer, and low fibrinogen	191	12		22		23.2		182	184	534
5	F	57	Venous thromboembolism on direct FXa inhibitors and elevated D-dimer	756	13		35		19.8		886	860	763
6	F	34	Cerebral ischemic events, normal coagulation studies	447	11		32		19.4		441	418	261
7	F	68	No relevant history, normal coagulation studies	326	9.6		31		22.8		337	389	348
8	F	68	Autoimmune/inflammatory disease, venous thromboembolism on warfarin, and elevated D-dimer and fibrinogen	611	14.8	12.4	28		22.9		885	777	3893
9	M	59	Cerebral ischemic events and elevated D-dimer	416	10.4		28		20.7		468	427	537
10	M	65	Cirrhosis, sepsis, and possible DIC	148	23.4	13.6	58	30	35.8	26	188	210	1585
11	F	39	Possible von Willebrand disease	230	10.3		33		22.2		247	222	<220
12	F	52	Venous thromboembolism on direct FXa inhibitors and elevated D-dimer	392	12.5		28		20.7		494	382	514
13	F	15	No relevant history, normal coagulation studies	298	11.5		28		22.8		350	319	<220
14	F	65	Cirrhosis and multiorgan failure	175	30.5	13.9	56	28	30.1	17.4	269	205	9742
15	M	56	Venous thromboembolism, multiple myeloma after stem cell transplant, possible dysfibrinogenemia vs fibrin polymerization defect, and intravascular coagulation fibrinolysis	169	13		22		35.8	27.2	276	274	65,940
16	M	37	Liver transplant, end-stage renal disease, and elevated D-dimer	275	11.6		32		26.8	17.3	320	319	533
17	F	46	Cirrhosis and coagulopathy	101	26	13.3	48	29	31.8	25.6	121	117	23,485
18	M	45	Venous thromboembolism on DTIs	378	10.2		34		69.5	18.2	358	387	<220
19	M	48	Pancreatitis, portal vein thrombosis, intravascular coagulation, and fibrinolysis	500	20.1	12.8	31		19.3		643	451	3484
20	F	44	Vitamin K deficiency	208	13.6		28		22.5		251	192	<220
21	M	23	Chronic DIC	140	15.1	12.5	28		26	17	156	135	10,605
22	M	44	Decompensated cirrhosis, intravascular coagulation, and fibrinolysis	166	18.2	12.2	39	32	22		221	185	2215
23	F	75	Therapy-related AML and DIC	151	15.1	12.1	30		29.6	23.8	194	175	26,527
24	F	40	Myocardial infarction status poststenting and heparin infusion	134	22.6	11.6	>400	274	>300	18.4	789	577	1203
25	F	83	DIC	51	15.6	12.1	25		30	30.7	56	73	12,555
26	M	34	Cirrhosis, intravascular coagulation, and fibrinolysis	121	36.8	13.8	75	32	31.3	31.5	171	136	20,247
27	F	79	DIC, venous thromboembolism, and acute liver disease	65	16	11	30		39	25	91	88	14,200
28	M	47	Antiphospholipid syndrome and venous thromboembolism on DTI	249	12.6		68	62	292	15	323	287	None
29	F	14	Congenital dysfibrinogenemia	64	14.3	12.3	33		33	41	443	444	<220
30	F	69	Compensated DIC 2/2 vasculitis	171	11.8		31		22		267	221	9540
31	M	78	Colon cancer and liver metastases	177	19.5	12.8	58	31	60.7	27.5	230	226	3775

Fibrinogen was measured by the Clauss functional assay, PT-Fib method, and immunoturbidimetric fibrinogen antigen assay; fibrinogen results are reported in mg/dL. Clinical history summarizes the indication for testing and relevant concurrent conditions, including liver disease, DIC, venous thromboembolism, ischemic events, inflammatory disease, and anticoagulant exposure, as applicable. Blank cells indicate that a mixing study was not performed or was not available. “<220” indicates a D-dimer value below the assay reporting limit, and “None” indicates that D-dimer was not available. aPTT normal range: <37 seconds; PT normal range: <12.5 seconds; D-dimer normal range: ≤500 ng/mL; TT normal range: <25 seconds; RT normal range: <24 seconds. If the PT mix or APTT mix is blank, it was not needed.

AML, acute myeloid leukemia; aPTT, activated partial thromboplastin time; DIC, disseminated intravascular coagulation; DTI, direct thrombin inhibitor; F, female; FXa, factor Xa; ID, identification number; M, male; PT, prothrombin time; PT-Fib, prothrombin time-derived fibrinogen; RT, reptilase time; TT, thrombin time.

### 2.3 Development and optimization of the LC-SRM method

Peptides for the candidate proteins were selected from a discovery analysis of plasma digests using previously described criteria [12], including peptides that are unique, devoid of any modifications or polymorphisms, and 7 to 25 amino acids long. The surrogate heavy isotope-labeled peptide standards were synthesized as previously described [13] using standard FMOC chemistry on a Liberty Blue peptide synthesizer (CEM Corporation), following the manufacturer’s instructions. The peptides contained a C-terminal lysine (^13^C_6_, ^15^N_2_) or arginine (^13^C_6_, ^15^N_4_), depending on the peptide sequence, and were >95% pure by high-performance liquid chromatography (HPLC). The lyophilized peptides were dissolved in 1 mL of a water/acetonitrile (70/30, v/v) mixture containing 0.1% formic acid. A stock solution of an equimolar mixture (10 μM) of synthetic peptide standards was prepared, aliquoted, and stored at −80 °C for future use.

The peptide standard mixture was analyzed in conventional data-dependent acquisition mode, as described previously [14], using an EASY-Spray UltiMate 3000 nanoLC connected to an Orbitrap Exploris 480 mass spectrometer (Thermo Fisher Scientific), and the highest-intensity tandem mass spectrometry (MS/MS) fragments were selected. Initially, 10 transitions per peptide were selected for collision energy optimization and LC-SRM method development on a TSQ Altis Triple Quadrupole mass spectrometer (Thermo Fisher Scientific) coupled to an Ultimate 3000 LC system (Thermo Fisher Scientific) [15]. The LC-SRM method was developed and optimized on an Accucore Vanquish C18+ UHPLC column (2.1 mm [ID] × 10 mm [length], 1.5 μm particle size; Thermo Fisher Scientific) maintained at 60 °C. The liquid chromatography method for standards, calibrators, and sample digests was set to 21 minutes at a flow rate of 300 μL/min, using the optimized gradient of solvent A (0.1% formic acid in water) and solvent B (0.1% formic acid in acetonitrile), starting at 3% B, ramping to 16% B at 14 minutes, to 90% B at 17 minutes, which was maintained until 20 minutes, then ramped down to 3% B at 21 minutes. A 3-minute column equilibration step was included at the start of the method. The TSQ Altis Triple Quadrupole mass spectrometer was operated in positive mode with the following source parameters: spray voltage (static): 3500 V; sheath gas: 35; auxiliary gas: 10; sweep gas: 1; ion transfer tube temperature: 350 °C; and vaporizer temperature: 320 °C. The selected reaction monitoring (SRM) parameters were set as follows: experiment type: SRM; chromatographic peak width: 10; collision gas pressure (mTorr): 2; cycle time (seconds): 0.83; Q1 resolution (full width at half maximum [FWHM]): 0.7; Q3 resolution (FWHM): 1.2. The source fragmentation and dwell time factor were set to zero and false, respectively.

### 2.4 Selection and characterization of target peptides to optimize the LC-SRM method for fibrinogen antigen detection

Surrogate peptides for total fibrinogen (α and γ chains) were selected from an in-house discovery analysis of plasma digests and publicly available datasets, including PeptideAtlas [16], and the Proteomic Assay Portal [17], and gnomAD [18]. The fibrinogen α and γ chain genes contain many single-nucleotide polymorphisms (SNPs) [19,20] that influence fibrinogen levels in individuals. Based on the gnomAD online database, peptides without SNPs were chosen for assay development. The selected peptide sequences were not part of D-dimer (for the γ chain) or fibrinopeptide A (for the α chain) and were unique to the fibrinogen chains (Figure 2A and Supplementary Figure S1). The selected peptides were synthesized using heavy isotope-labeled C-terminal lysine or arginine FMOC chemistry on a Liberty Blue peptide synthesizer. The peptides were subjected to collision energy optimization to enhance detection. First, the default collision energy data were exported from Skyline version 23.1 [21] using the TSQ Altis Triple Quadrupole mass spectrometer’s default collision energy linear equation for doubly and triply charged peptides as an unscheduled method [22]. Next, SRM data acquisition for synthetic standard peptides was performed. Peptides were then scheduled, and collision energies at 5 distinct steps on either side of the anticipated default value for each transition were exported. The acquired LC-SRM data were imported into Skyline version 23.1 [21] to determine the updated collision energy equation for future experiments. This process was repeated in duplicate at the same peptide concentration until the maximum intensity for each fragment was reached. The Clinical and Laboratory Standards Institute C62-A guideline [23] recommends using 1 qualifier and 1 quantifier transition in clinical liquid chromatography-tandem mass spectrometry (LC-MS/MS) methods to ensure the identification and quantification of target analytes with adequate specificity. However, to further improve the robustness and reliability of our quantification, we used an average of more than 3 transitions per peptide (Supplementary Table S1 and Supplementary Figure S2C).

**Figure 2:**
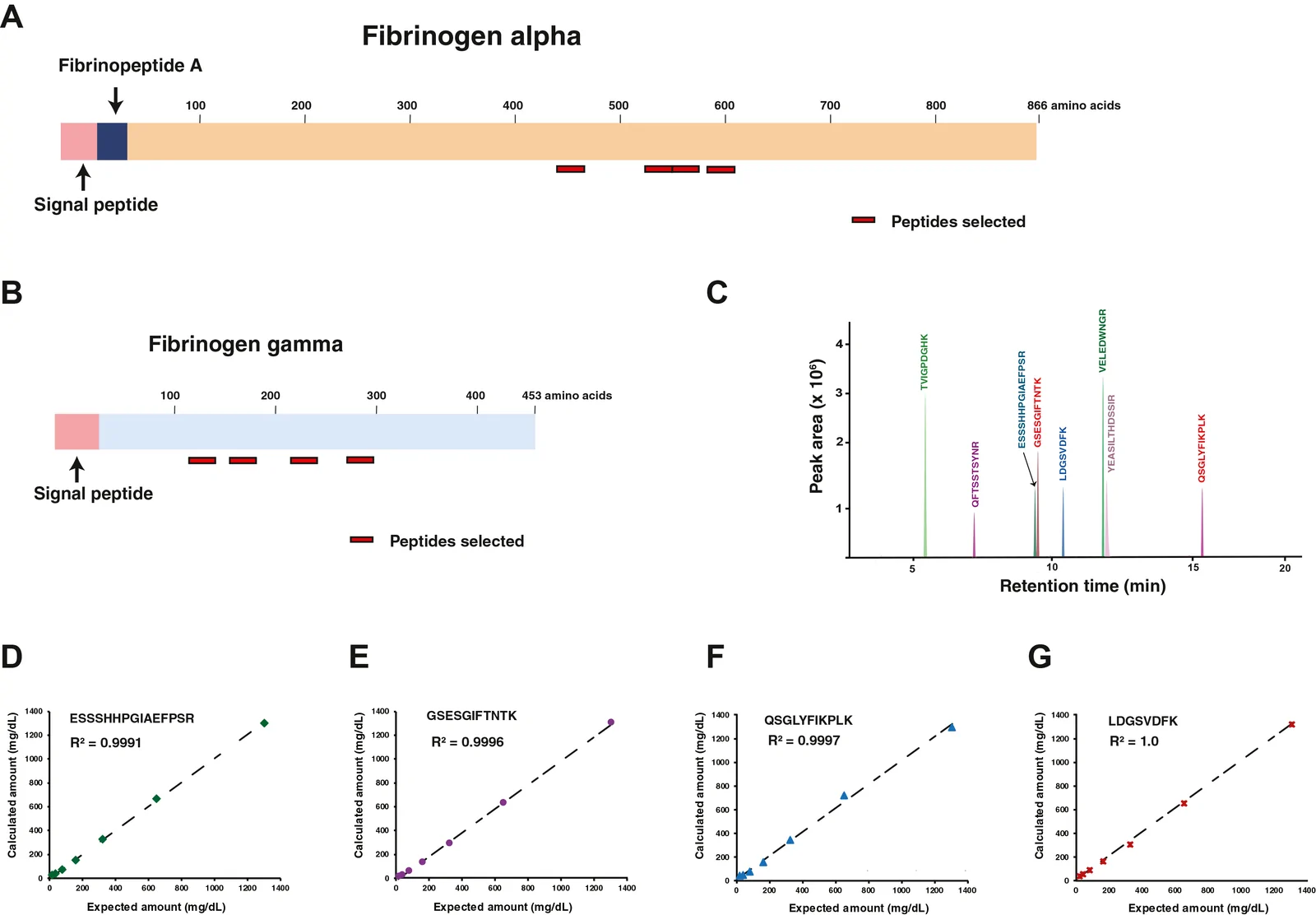
Selection and quantitative performance of fibrinogen peptides for targeted liquid chromatography-mass spectrometry (LC-MS) analysis. (A) Schematic representation of the fibrinogen α chain showing the signal peptide, fibrinopeptide A region, and the locations of the selected proteotypic peptides. (B) Schematic representation of the fibrinogen γ chain indicating the signal peptide and selected peptides used for analysis. Red bars denote peptides selected for targeted quantification. (C) Extracted ion chromatogram of the 8 selected peptides from the α and γ chains of fibrinogen. (D–G) Linearity assessment comparing expected vs calculated fibrinogen concentrations using individual peptides (ESSSHHPGIAEFPSR, GSESGIFTNTK, QSGLYFIKPLK, and LDGSVDFK). Excellent linearity was observed across the tested concentration range of 10.2 mg/dL to 1307.5 mg/dL (r^2^ ≥ .99).

### 2.5 Calibration curve determination reproducibility and precision studies

Liquid chromatography conditions were optimized using synthetic isotopically labeled internal standard peptides. CRYOcheck Normal Reference Plasma, with a fibrinogen level of 327 mg/dL determined by the Clauss assay, was used to prepare external calibrators ranging from 10.2 mg/dL to 1307.5 mg/dL by diluting the lyophilized reference plasma with Owren-Koller buffer. Calibrators were prepared fresh for each experimental run and discarded after use. CRYOcheck Abnormal 1 Reference Control was used for quality control (QC) samples at 3 fibrinogen levels: low (52-63 mg/dL), medium (104-127 mg/dL), and high (208-254 mg/dL). Intra- and interday precision was determined by analyzing 3 QC samples over 3 consecutive runs within a single day for intraday precision and 3 replicates per day for 5 days for interday precision. Method precision was expressed as the coefficient of variation (CV; %) and percent recovery relative to the assigned QC level.

### 2.6 Statistical analysis

Peaks were integrated in Skyline version 23.1 [21] and examined for accurate, consistent selection of both endogenous and synthetic standard peptides across samples. The calibration curve was used to calculate endogenous peptide concentrations in samples based on the peak area ratio of endogenous to internal standard peptides. The integrated data were exported to Excel (Microsoft Corporation) for further quantification. Statistical analysis was performed in GraphPad Prism (version 10.0.0).

### 2.7 Method comparison and interference studies

A normal donor study was conducted using plasma samples from 47 healthy individuals to establish a reference interval for the LC-SRM method. The samples were also analyzed by the Clauss assay and PT-Fib method to compare results across methods. A method comparison was performed using 31 blinded clinical samples. Samples were categorized by Clauss fibrinogen as low (<150 mg/dL), normal, or high (>400 mg/dL). All samples were analyzed by the Clauss assay and PT-Fib method using the HemosIL Fibrinogen-C Kit and HemosIL PT-Fibrinogen Kit (Werfen), respectively, on an ACL TOP analyzer (Werfen). A subset of samples was analyzed by immunoturbidimetry for fibrinogen antigen concentration (Mayo Clinic's Cardiology Special Laboratory). Fibrinogen was also assessed by the Clauss assay to determine thrombin-coagulable fibrinogen; Clauss activity, PT-Fib, PT, PT 1:1 immediate mixing studies, aPTT, aPTT 1:1 immediate mixing studies, and D-dimer were included, where available, to provide clinical context for interpretation of the assay. Method comparison studies included percent agreement calculations for categorical classifications (low, normal, or high) and correlation analyses among methods.

Interference studies evaluated the impact of fibrinolysis and direct thrombin inhibition on fibrinogen measurements. For fibrinolysis interference, 5 normal donor plasma samples were treated with streptokinase. Each sample was analyzed before and after treatment using the Clauss assay, PT-Fib, D-dimer, and fibrinogen antigen by immunoturbidimetry. The same set of samples was analyzed by LC-SRM to quantify fibrinogen using the GSESGIFTNTK and QSGLYFIKPLK peptides. Correlation analysis was performed between changes in antigen measurements and streptokinase treatment. The effect of direct thrombin inhibition was assessed by adding bivalirudin at 3 concentrations (0.5, 1.0, and 2.0 ng/mL) to normal donor plasma. These samples were analyzed by all methods, including fibrinogen antigen by immunoturbidimetry, and the results were evaluated for concentration-dependent effects for each measurement method.

### 2.8 Ethics

This study was approved by the Mayo Clinic Institutional Review Board (Institutional Review Board number 22-006836) and conducted in accordance with the Declaration of Helsinki and applicable regulations. All participants, or their legal guardians, as applicable, provided written informed consent prior to enrollment and sample collection.

## 3 Results

### 3.1 Development of an SRM-based targeted mass spectrometric assay

Surrogate peptides for fibrinogen (α and γ chains) were selected based on plasma digest runs and public resources (PeptideAtlas and the Proteomic Assay Portal), ensuring exclusion of peptides with known SNPs (gnomAD) and those corresponding to D-dimer (γ chain) or fibrinopeptide A. Initially, 4 candidate peptides for the α chain (TVIGPDGHK, GSESGIFTNTK, ESSSHHPGIAEFPSR, and QFTSSTSYNR) and the γ chain (LDGSVDFK, VELEDWNGR, YEASILTHDSSIR, and QSGLYFIKPLK) were selected for quantitation (Figure 2A, B). Sequence alignment of the fibrinogen α and γ chains confirmed that the selected peptides were unique to each chain, ensuring specificity (Supplementary Figure S1). For these peptides, heavy isotope-labeled peptides were synthesized and used to optimize LC-SRM parameters, including selecting the top 4 performing transitions for each peptide. LC-SRM data were iteratively analyzed in Skyline version 23.1 [21] until maximum fragment ion intensity was achieved, and the top 4 most intense transitions per peptide were selected for precise quantification (Supplementary Table S1 and Supplementary Figure S2C). The liquid chromatography gradient was optimized to separate the peptides efficiently, minimize ion suppression, and maximize signal-to-noise ratios (Figure 2C).

### 3.2 Analytical performance

Initial evaluation of the LC-SRM assay demonstrated linearity (r^2^ > .98) across the measurement range of 10.2 to 1307.5 mg/dL for all validated peptides (Figure 2D–G and Supplementary Table S3). Of the initial 8 peptides, 2 for the α chain (TVIGPDGHK and QFTSSTSYNR) and 2 for the γ chain (YEASILTHDSSIR and LDGSVDFK) were not pursued further because of inconsistent transition ratios, signal instability (Supplementary Figure S2A, B), and high CVs (>20%), especially at lower concentrations. The reliability of this quantification approach was further validated through intraday and interday precision assessments, with CV values below 20% across all QC levels (Table 2). Interday CVs ranged from 3.15% to 14.63%. The highest variability was observed for QSGLYFIKPLK (13.24% at medium QC) and LDGSVDFK (14.63% at medium QC), but still within the acceptance criteria. Carryover was evaluated to ensure the accuracy and reliability of the analytical method. This was accomplished by running a high-concentration calibrator 3 consecutive times to saturate the system. Immediately following the calibrator runs, 3 consecutive diluent blanks were analyzed to detect any residual analyte carryover from the high-concentration samples. The mean background signal in the blanks was <5 mg/dL, below the limit of detection signal for both peptides considered for quantification, confirming no carryover across samples. Retention time alignment across calibrators and QC samples for the internal standard and analyte was precise (Supplementary Table S4). In addition, N-terminal glutamine in the γ-chain QSGLYFIKPLK peptide is known to form pyroglutamate during ionization, depending on the source voltage used in the analysis [24]. We systematically characterized the extent of pyroglutamate formation in the analysis. For this purpose, pooled plasma samples were analyzed to obtain pyroglutamate-specific fragments (Supplementary Figure S3A), and these were further used to quantify the percent pyroglutamate formation by LC-SRM, which was <5% across the calibrants used (mean, 4.4%; Supplementary Figure S3B and Supplementary Table S8). The final method incorporated 2 peptides per chain; however, quantification was performed using the GSESGIFTNTK peptide for the α chain and the QSGLYFIKPLK peptide for the γ chain, since these peptides exhibited minimal percent difference in fibrinogen for the normal samples; based on these criteria, the α chain peptide was considered to represent total fibrinogen levels.

**Table 2:** Method performance of fibrinogen peptides by liquid chromatography–selected reaction monitoring (LC-SRM).

Peptide sequence	Assigned value by the Clauss assay (mg/dL)	Obtained value by LC-SRM (mg/dL)	Percent deviation from assigned value (%)	Intraday CV (%)	Interday CV (%)
**GSESGIFTNTK**	Low QC (52-63)	55.52	−3.87	0.21	7.50
	Medium QC (104-127)	97.94	−15.20	0.71	9.74
	High QC (208-254)	184.25	−20.24	2.61	3.45
**ESSSHHPGIAEFPSR**	Low QC (52-63)	54.48	−5.66	0.63	8.61
	Medium QC (104-127)	95.79	−17.06	1.93	10.44
	High QC (208-254)	181.96	−21.23	1.63	3.15
**QSGLYFIKPLK**	Low QC (52-63)	62.16	7.64	7.17	13.06
	Medium QC (104-127)	104.72	−9.33	5.49	13.24
	High QC (208-254)	205.56	−11.01	1.49	10.91
**LDGSVDFK**	Low QC (52-63)	56.86	−1.55	2.21	10.23
	Medium QC (104-127)	97.20	−15.84	1.88	14.63
	High QC (208-254)	187.59	−18.79	1.74	3.35

The table summarizes the assigned concentrations (mg/dL), measured concentrations (mg/dL), percent recovery, and intra- and interday CVs (%) for the 4 target peptides (GSESGIFTNTK, ESSSHHPGIAEFPSR, QSGLYFIKPLK, and LDGSVDFK). QC levels were assessed at low (52-63 mg/dL), medium (104-127 mg/dL), and high (208-254 mg/dL) concentration ranges. Recovery was calculated as the deviation (%) of the obtained values from the assigned values.

CV, coefficient of variation; LC-SRM, liquid chromatography-selected reaction monitoring; QC, quality control.

### 3.3 Normal donor study

Analysis of 47 normal donor samples established a reference distribution of fibrinogen measurements by LC-SRM. Fibrinogen antigen concentrations followed a Gaussian distribution, with a mean of 315 mg/dL (SD, 42 mg/dL) for the α chain and 298 mg/dL (SD, 38 mg/dL) for the γ chain (Figure 3A and Supplementary Table S5). Two samples (4%) showed elevated levels above 400 mg/dL for both chains, which were subsequently confirmed by the Clauss and PT-Fib assays. The α-to-γ chain ratios in normal donors had a mean of 1.06 (95% CI, 0.98-1.14). A subset of normal samples (*n* = 20) was analyzed by immunoturbidimetry for fibrinogen antigen, showing good correlation, with a Pearson correlation coefficient (r) of .762.

**Figure 3:**
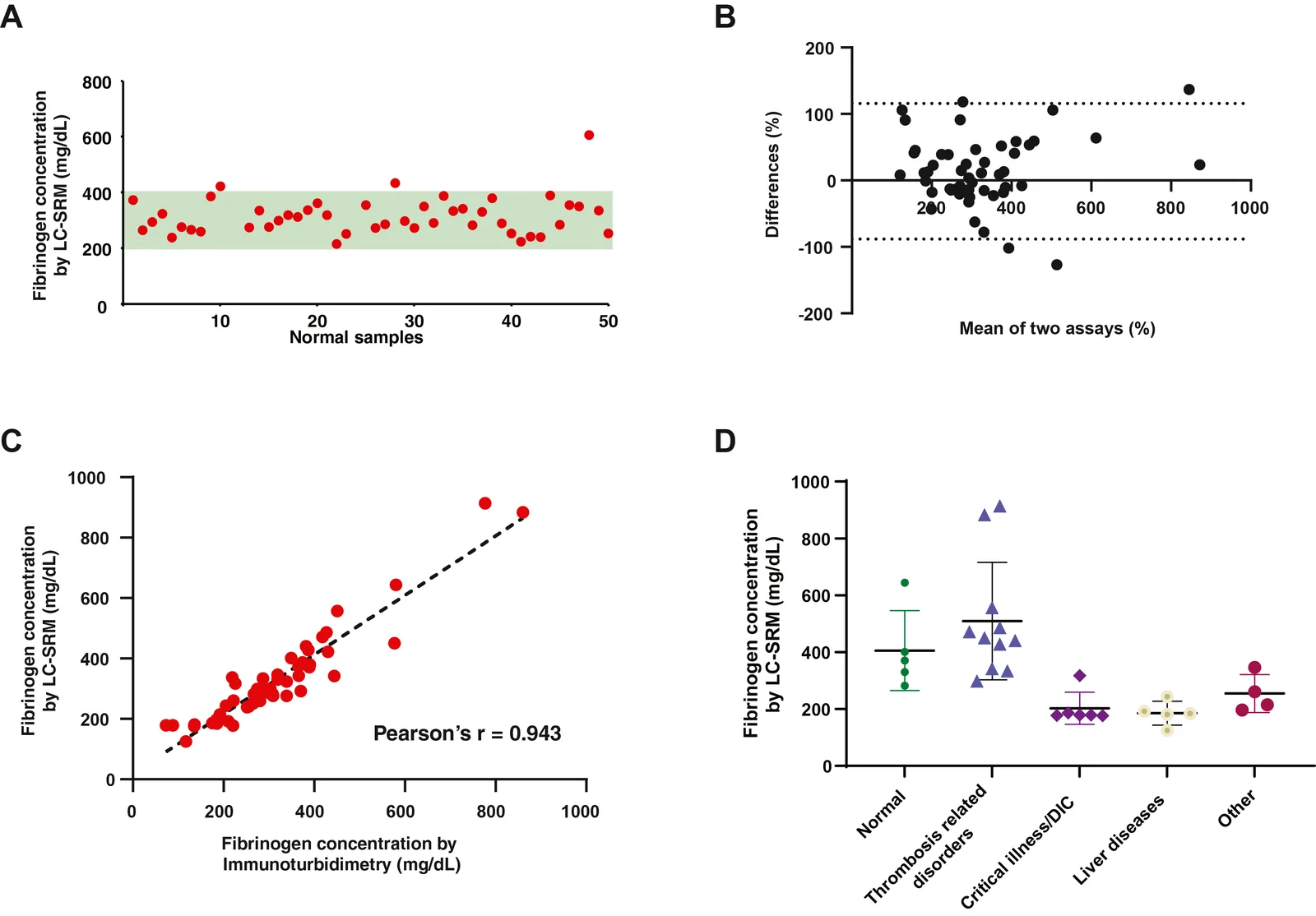
Validation of liquid chromatography-selected reaction monitoring (LC-SRM) fibrinogen measurements. (A) LC-SRM assay results for normal samples. The fibrinogen α- and γ-chain concentrations were determined in normal donor plasma samples, and values fell within the expected range for all samples analyzed (200-400 mg/dL). (B) Bland–Altman analysis comparing the LC-SRM method with the immunoturbidimetric assay for fibrinogen measurement. Differences between the 2 assays (%, y-axis) are plotted against the mean of the paired values (x-axis). The central line denotes the mean bias, while dotted lines indicate the 95% limits of agreement. (C) Fibrinogen antigen levels measured by LC-SRM showed a strong correlation with immunoturbidimetry results in blinded samples (Pearson’s correlation coefficient [r] = .983). (D) Grouped comparison of fibrinogen levels measured by LC-SRM vs immunoturbidimetry across various clinical categories (normal, thrombosis-related disorders, critical illness/disseminated intravascular coagulation [DIC], liver diseases, and others).

### 3.4 Method comparison

Fibrinogen levels were measured in 31 clinical samples and compared with those from commonly used functional assays. Of the participants, 17 were female, and 14 were male, with ages ranging from 15 to 83 years (Table 1 and Supplementary Table S6). Targeted LC-SRM quantification yielded comparable values, with a mean fibrinogen concentration of 348 mg/dL. The overall fibrinogen levels in all 31 clinical samples and 20 normal samples analyzed by immunoturbidimetric assay showed a strong correlation, with Pearson’s r = .943 (Figure 3C and Supplementary Tables S6 and S7). Agreement between the immunoturbidimetric and LC-SRM assays was evaluated using Bland–Altman analysis. The mean difference (bias) was −13.9 units, indicating that fibrinogen values obtained by the immunoturbidimetric assay were, on average, slightly lower than those obtained with LC-SRM. The 95% limits of agreement ranged from −115.8 to +88.1, showing that most paired measurements fell within this interval (Figure 3B). A paired *t*-test confirmed that the mean difference was not statistically significant (*P* > .05), suggesting no systematic deviation between the 2 assays. Clinical sample features were determined and compared with LC-SRM data. When stratified by clinical category, distinct group-specific patterns were observed (Figure 3D). A venous thromboembolism sample from a patient (sample ID: 5) receiving a direct FXa inhibitor showed marked hyperfibrinogenemia across all methods, with a Clauss fibrinogen of 756 mg/dL, PT-Fib of 886 mg/dL, immunoturbidimetric fibrinogen of 860 mg/dL, and an LC-SRM value of 883.4 mg/dL. Similarly, a sample from a patient with autoimmune/inflammatory disease and venous thromboembolism on warfarin (sample ID: 8) showed high fibrinogen by the Clauss assay (611 mg/dL), PT-Fib (885 mg/dL), immunoturbidimetry (777 mg/dL), and LC-SRM (913.8 mg/dL). These results demonstrate that LC-SRM fibrinogen peptide measurements track antigen-based measurements in samples with elevated fibrinogen. Five samples had intermediate LC-SRM concentrations and were categorized as normal. These samples, with no relevant clinical history or normal coagulation studies, showed overall agreement among functional, derived, antigen-based, and LC-SRM measurements. One sample (sample ID: 2), with elevated D-dimer and fibrinogen but no other relevant clinical history, showed increased Clauss fibrinogen (556 mg/dL), PT-Fib (666 mg/dL), immunoturbidimetric fibrinogen (580 mg/dL), and an LC-SRM value (643.8 mg/dL).

The lowest fibrinogen concentrations were observed in patients with critical illness or disseminated intravascular coagulation (DIC), ranging from 177 to 317 mg/dL (Figure 3D). Samples from patients with liver disease, cirrhosis, sepsis, or multiorgan dysfunction showed reduced fibrinogen levels and broader abnormalities in global coagulation tests. A sample from a patient (sample ID: 10) with cirrhosis, sepsis, and possible DIC showed a low Clauss fibrinogen of 148 mg/dL, PT-Fib of 188 mg/dL, immunoturbidimetric fibrinogen of 210 mg/dL, and an LC-SRM value of 192.2 mg/dL, along with prolonged PT (23.4 seconds), prolonged aPTT (58 seconds), and an elevated D-dimer (1585 ng/mL). Another sample from a patient (sample ID: 14) with cirrhosis and multiorgan failure showed a Clauss fibrinogen of 175 mg/dL, PT-Fib of 269 mg/dL, immunoturbidimetric fibrinogen of 205 mg/dL, and an LC-SRM value of 244.0 mg/dL, with markedly prolonged PT (30.5 seconds) and aPTT (56 seconds) and a D-dimer of 9742 ng/mL. Another sample from a patient (sample ID: 17) with cirrhosis and coagulopathy showed low fibrinogen by all routine methods, with a Clauss fibrinogen of 101 mg/dL, PT-Fib of 121 mg/dL, immunoturbidimetric fibrinogen of 117 mg/dL, and an LC-SRM value of 125.1 mg/dL. These findings are consistent with reduced fibrinogen production, increased consumption, and systemic coagulation activation in advanced liver disease and critical illness. Samples from patients with cerebral ischemic events showed preserved fibrinogen concentrations and limited assay discordance. One sample (sample ID: 6) showed a Clauss fibrinogen of 447 mg/dL, PT-Fib of 441 mg/dL, immunoturbidimetric fibrinogen of 418 mg/dL, and an LC-SRM value of 471.6 mg/dL. Another ischemic event sample showed a Clauss fibrinogen of 416 mg/dL, PT-Fib of 468 mg/dL, immunoturbidimetric fibrinogen of 427 mg/dL, and an LC-SRM value of 486.2 mg/dL. These samples did not show the pronounced activity-antigen discordance observed in dysfibrinogenemia or anticoagulant-associated samples. Patients with hematological disorders (*n* = 6) showed fibrinogen levels ranging from 298 to 450 mg/dL. Finally, the single patient with colon cancer and liver metastases exhibited markedly decreased fibrinogen values, clustering with the liver disease group. Association of LC-SRM values for the α chain with the Clauss assay (Supplementary Figure S4A) and the PT-Fib assay (Supplementary Figure S4C) showed excellent correlation (r = .91 and r = .93, respectively). Additionally, LC-SRM levels for the γ chain correlated well with the Clauss assay (r = .90; Supplementary Figure S4B) and the PT-Fib assay (r = .92; Supplementary Figure S4D). Overall, category-based analysis demonstrated that LC-SRM fibrinogen measurements generally paralleled immunoturbidimetric antigen results and provided additional support for antigen preservation or depletion across diverse clinical categories. The inclusion of PT, aPTT, mixing studies, D-dimer, and LC-SRM fibrinogen measurements provides clinically relevant context for interpreting the fibrinogen assay.

### 3.5 Interference studies

The effect of fibrinolysis on fibrinogen measurements was evaluated in 5 normal donor plasma samples treated with streptokinase (Table 3). Streptokinase treatment led to marked reductions in Clauss fibrinogen (>75% decrease) and substantial increases in D-dimer (>5000 fibrinogen equivalent units) across all samples, confirming effective fibrinolysis. Despite these changes in functional measurements, LC-SRM antigen measurements remained relatively stable. Mean changes in antigen levels after streptokinase treatment for the GSESGIFTNTK and QSGLYFIKPLK peptides were −14.2% and −7.9%, respectively, with a moderate correlation between posttreatment reductions in fibrinogen antigen measurements and streptokinase (r = .48 and r = .43 for GSESGIFTNTK and QSGLYFIKPLK peptides, respectively).

**Table 3:** Interference studies with streptokinase treatment.

Sample ID	FibAg (mg/dL)	Clauss Fib (mg/dL)	PT-Fib (mg/dL)	D-dimer (FEU)	Fibrinogen α (mg/dL)	Fibrinogen γ (mg/dL)
SK 01	319	<25	<60	9625	314.4	372.3
Untreated 01	376	374	NP	218	387.0	390.2
SK 02	300	39	222	6052	363.6	387.2
Untreated 02	314	308	NP	220	272.8	329.7
SK 03	387	74	274	6708	373.2	450.4
Untreated 03	415	392	NP	525	426.3	447.7
SK 04	339	74	232	5332	314.3	383.8
Untreated 04	358	321	NP	106	381.6	381.6
SK 05	327	85	63	5867	311.6	353.8
Untreated 05	343	325	NP	628	363.9	380.5
Biv_2.0 ng/mL	315	151	354	NP	256.3	307.2
Biv_1.0 ng/mL	313	231	314	NP	269.0	301.0
Biv_0.5 ng/mL	315	278	311	NP	227.1	278.7

Interference studies were performed after treating normal donor plasma with SK and spiking it with different concentrations of Biv, a direct thrombin inhibitor.

Biv, bivalirudin; Clauss Fib, Clauss fibrinogen; FEU, fibrinogen equivalent units; FibAg, fibrinogen antigen; NP, not performed; PT-Fib, prothrombin time-derived fibrinogen; SK, streptokinase-treated.

The impact of direct thrombin inhibition was also assessed by treating normal donor plasma with increasing concentrations of the anticoagulant bivalirudin (0.5, 1.0, and 2.0 ng/mL), which binds thrombin directly. Bivalirudin treatment caused a concentration-dependent decrease in fibrinogen measured by the Clauss assay (278, 231, and 151 mg/dL, respectively, compared with a baseline of 325 mg/dL) and a false increase in PT-Fib at the highest bivalirudin concentration. In contrast, LC-SRM antigen and fibrinogen antigen measured by immunoturbidimetry remained stable across all bivalirudin concentrations. The GSESGIFTNTK and QSGLYFIKPLK peptide measurements varied by <15% across all bivalirudin concentrations.

## 4 Discussion

The LC-SRM method described in this study demonstrates several key advantages for fibrinogen measurement in clinical samples. In normal donor samples, the method showed excellent agreement with existing assays, yielding a reference interval consistent with fibrinogen measurements by the Clauss assay. When fibrinogen levels from several test samples across different disease types were compared with widely used functional assays (Clauss and PT-derived assays, PT, aPTT, mixing studies, and D-dimer), LC-SRM showed strong correlations (r = .90-.93) for both α and γ chains. Importantly, LC-SRM accurately captured the spectrum of fibrinogen concentrations across diverse clinical samples, including elevated levels in thrombosis-related disorders and reduced concentrations in patients with DIC or advanced liver disease. This indicates that the method accurately quantifies circulating fibrinogen in samples without interfering substances, while offering the additional capability of measuring individual chain concentrations.

A significant advantage of the LC-SRM method is its robustness to DTIs. While Clauss fibrinogen showed the expected concentration-dependent decrease with increasing bivalirudin levels [8], LC-SRM measurements remained stable. This finding is particularly important given the use of DTIs in clinical practice and their known interference with functional fibrinogen assays [8,10]. The ability to accurately measure fibrinogen in patients addresses an important clinical need, especially in the setting of cardiac surgery for patients with heparin-induced thrombocytopenia who require anticoagulation with a DTI and accurate measurement to guide replacement and antifibrinolytic drugs.

The method’s performance in the presence of FDPs requires further optimization. While LC-SRM measurements showed good correlation with immunological fibrinogen assays across samples, both methods are currently unable to discriminate between intact fibrinogen and FDPs. This limitation is well documented for immunological methods due to antibody cross-reactivity with degradation products [6,9]. Future refinements of the LC-SRM method could incorporate a preanalytical separation step using molecular-weight cutoff filters to exclude low-molecular-weight FDPs. This modification could potentially provide more accurate measurement of intact fibrinogen in samples with elevated FDPs, such as in DIC.

The LC-SRM method offers several unique capabilities that complement existing fibrinogen assays. Unlike traditional methods that rely on optical detection and are susceptible to interference from hemolysis, icterus, and lipemia [10,25], mass spectrometry-based detection is unaffected by these matrix effects. Additionally, the ability to measure α and γ chains independently could provide new insights into fibrinogen variants and chain-specific abnormalities. Areas for future development include streamlining the sample preparation workflow for routine clinical implementation and expanding the peptide panel to include the β chain. Validation studies in specific clinical scenarios, such as dysfibrinogenemia, where both antigen and activity measurements are crucial for diagnosis [6], would help define the optimal role of this assay in the diagnostic algorithm.

In conclusion, the LC-SRM method is a valuable addition to the existing repertoire of fibrinogen assays. Its ability to accurately measure fibrinogen in samples containing DTIs addresses an important clinical need. Although further validation is warranted, this method shows promise as a reference measurement procedure and could also provide new insights into fibrinogen disorders through chain-specific quantification.

## Supplementary material

Supplementary Figures S1-S4 Supplementary Figure 1. Amino acid alignment of the fibrinogen α and γ chain.. The highlighted (in red dotted square) peptides selected for the antigen measurement were unique to the chain.Extracted chromatograms of initially selected fibrinogen peptides α and γ peptides. Out of 4 peptides selected, 2 peptides each for the α chain (TVIGPDGHK and QFTSSTSYNR, (A) and γ chain (YEASILTHDSSIR and LDGSVDFK, (B) showed distorted signal at the lower concentration (10.2 mg/dL) and hence excluded from further analysis.Supplementary Figure 2. Extracted chromatograms of initially selected fibrinogen peptides α and γ peptides. Out of 4 peptides selected, 2 peptides each for the α chain (TVIGPDGHK and QFTSSTSYNR, (A) and γ chain (YEASILTHDSSIR and LDGSVDFK, (B) showed distorted signal at the lower concentration (10.2 mg/dL) and hence excluded from further analysis.Supplementary Figure 3. Characterization and quantification of the pyroglutamate modified peptide. Tandem mass spectra of modified pyro-QSGLYFIKPLK confirming sequence identity (A). The 4 top fragments were considered to measure the level of the pyro modified version of the QSGLYFIKPLK peptide by LC-SRM compared with native version across the calibrators (A-H). The total percentage amount of pyro-peptide was below 5% (mean, 4.4).Supplementary Figure 4. Scatter plots show pairwise comparisons of fibrinogen concentrations measured by LC-SRM against functional assays. (A) Fibrinogen α chain measured by LC-SRM compared with Clauss assay (Pearson’s r = 0.91). (B) Fibrinogen γ chain measured by LC-SRM compared with Clauss assay (Pearson’s r = 0.90). (C) Fibrinogen α chain measured by LC-SRM compared with PT-derived fibrinogen (Pearson’s r = 0.93). (D) Fibrinogen γ chain measured by LC-SRM compared with PT-derived fibrinogen (Pearson’s r = 0.92). Each dot represents an individual clinical sample. The high correlation values demonstrate strong agreement between LC-SRM-based quantification and traditional clot-based assays

Supplementary Table S1 The details of the peptides selected and SRM parameters. Isotope labeled peptides standards were analyzed on Orbitrap to select precursor and fragments. SRM parameter optimization included collision energy optimization for these peptides for each transition and top transitions were selected based on their performance during the optimization.

Supplementary Table S2 Summary of the results obtained from duplicate amino acid analyses of the synthetic stable isotope–labeled (SIL) peptides corresponding to Fibrinogen α and γ chain is presented.

Supplementary Table S3 Coefficient of determination (r^2^) for calibration curves is shown for 2 α-chain peptides (GSESGIFTNTK, ESSSHHPGIAEFPSR) and 2 γ-chain peptides (LDGSVDFK, QSGLYFIKPLK) across 5 independent days (Day 1–Day 5). Linearity was evaluated over the entire analytical range (10.2–1308 mg/dL) and within the low-concentration range (10.2–327 mg/dL).

Supplementary Table S4 Retention time differences for calibrators and QCs samples. The retention time difference between the analytes and IS should be within ±0.1 min.

Supplementary Table S5 Demographic characteristics of the normal samples included 47 participants (24 males and 26 females) with ages ranging from 22 to 83 years.

Supplementary Table S6 Demographic characteristics of the test samples. The test samples included samples across age group 15 to 83 years. These samples were analyzed by Clauss assay, PT-derived fibrinogen assay and Immunoturbidimetry assay. The LC-SRM analysis of fibrinogen α and γ chains yielded comparable results with excellent correlation.

Supplementary Table S7 A subset of normal-donor samples was tested in parallel by Clauss, PT-derived fibrinogen (PT-Fib), and immunoturbidimetry (all reported in mg/dL). For each sample, the table lists age, gender, and LC-SRM-derived concentrations for the fibrinogen α and γ chains. To evaluate intermethod agreement, 2 percentage metrics are provided: Clauss/PT-Fib % difference and Clauss/Immunoturbidimetry % difference.

Supplementary Table S8 Peak areas are shown for the pyroglutamate form (pyro-QSGLYFIKPLK, m/z 426.24+++) and the native peptide (QSGLYFIKPLK, m/z 431.92+++). Zero values indicate no detectable pyro form. The mean fraction of pyroglutamate formation across measurements was 4.4%.
