# Supplementary material for: A targeted mass spectrometric assay for quantitative detection of fibrinogen in plasma
Source: Res Pract Thromb Haemost. 2026 Sep 18;10(7):106974. doi: 10.1016/j.rpth.2026.106974 (PMC13744374; doi:10.1016/j.rpth.2026.106974)

Supplementary Figure S1

```
Fibrinogen alpha    MFSMRIVCLVLSVVGTAWTADSGEGDFLAEGGGVRGPRVVERHQSACKDSDWPFCSDEDW    60
Fibrinogen gamma    ------------------------------------------------------------

Fibrinogen alpha    NYKCPSGCRMKGLIDEVNQDFTNRINKLKNSLFEYQKNNKDSHSLTTNIMEILRGDFSSA    120
Fibrinogen gamma    ------------------------------------------------------------

Fibrinogen alpha    NNRDNTYNRVSEDLRSRIEVLKRKVIEKVQHIQLLQKNVRAQLVDMKRLEVDIDIKIRSC    180
Fibrinogen gamma    ------------------------------------------------------------

Fibrinogen alpha    RGSCSRALAREVDLKDYEDQQKQLEQVIAKDLLPSRDRQHLPLIKMKPVPDLVPGNFKSQ    240
Fibrinogen gamma    ------------------------------------------------------------

Fibrinogen alpha    LQKVPPEWKALTDMPQMRMELERPGGNEITRGGSTSYGTGSETESPRNPSSAGSWNSGSS    300
Fibrinogen gamma    ------------------------------------------------------------

Fibrinogen alpha    GPGSTGNRNPGSSGTGGTATWKPGSSGPGSTGSWNSGSSGTGSTGNQNPGSPRPGSTGTW    360
Fibrinogen gamma    ------------------------------------------------------------

Fibrinogen alpha    NPGSSERGSAGHWTSESSVSGSTGQWHSESGSFRPDSPGSGNARPNNPDWGTFEEVSGNV    420
Fibrinogen gamma    -----------------------------------------------MSWS--------L    5
                                                                    .*.        :

Fibrinogen alpha    SPGTRREYHTEKLVTSKGDKELRTGKEKVTSGSTTTTRRSCSKTVTKTVIGPDGHKEVTK    480
Fibrinogen gamma    HPRNLILYFYALLFLSSTC------------VAYVATRDNC-------------------    34
                     * .   *.   *. *.               : .:** .*

Fibrinogen alpha    EVVTSEDGSDCPEAMDLGTLSGIGTLDGFRHRHPDEAAFFDTASTGKTFPGFFSPMLGEF    540
Fibrinogen gamma    CILDERFGSYCPTTCGIADFLS-----TYQTKVDKD------------L-----QSLEDI    72
                     :: .. ** ** : .:. : .      :: :  .:            :       * ::

Fibrinogen alpha    VSETESRGSESGIFT-----NTKESSSHHPGIAE---FPSRGKS---SSYSKQF-TSSTS    588
Fibrinogen gamma    LHQVENKTSEVKQLIKAIQLTYNPDESSKPNMIDAATLKSRKMLEEIMKYEASILTHDSS    132
                    : :.*.: **   :      . : ..* :*.: :   : **       .*. .: * .:*

Fibrinogen alpha    YNRGDSTFESKSYKMADEAGSEADHEGT---HSTKRGHAKSRPVRDCDDVLQTHPSGTQS    645
Fibrinogen gamma    IRYLQEIYNSNNQKIVNLKEKVAQLEAQCQEPCKDTVQIHDITGKDCQDIA--NKGAKQS    190
                     .  :. ::*:. *:.:   . *: *.     ...  : :.   :**:*:   : ...**

Fibrinogen alpha    GIFNIKLPGSSKIFSVYCDQETSLGGWLLIQQRMDGSLNFNRTWQDYKRGFGSLNDEGEG    705
Fibrinogen gamma    GLYFIKPLKANQQFLVYCEIDGSGNGWTVFQKRLDGSVDFKKNWIQYKEGFGHLSPTGTT    250
                    *:: **   :.: * ***: : * .** ::*:*:***::*::.* :**.*** *.  *

Fibrinogen alpha    EFWLGNDYLHLLTQRGS---VLRVELEDWAGNEAYAEYH-FRVGSEAEGYALQVSSYEGT    761
Fibrinogen gamma    EFWLGNEKIHLISTQSAIPYALRVELEDWNGRTSTADYAMFKVGPEADKYRLTYAYFAGG    310
                    ******: :**:: :.:   .******** *. : *:*  *:** **: * *  : : *

Fibrinogen alpha    AGDAL----IEGSVEEGAEYTSHNNMQFSTFDRDADQWEENCAEVYGGGWWYNNCQAANL    817
Fibrinogen gamma    DAGDAFDGFDFGDDPSDKFFTSHNGMQFSTWDNDNDKFEGNCAEQDGSGWWMNKCHAGHL    370
                     ..        *.  ..  :****.*****:*.* *::* ****  *.*** *:*:*.:*

Fibrinogen alpha    NGIYYPGGSYDPRNNSPYEIENGVVWVSFRGADYSLRAVRMKIRPLVTQ-----------    866
Fibrinogen gamma    NGVYYQGGTYSKA-STPNGYDNGIIWATWKTRWYSMKKTTMKIIPFNRLTIGEGQQHHLG    429
                    **:** **:*.   .:*   :**::*.:::   **:: . *** *:

Fibrinogen alpha    ------------------------     866
Fibrinogen gamma    GAKQVRPEHPAETEYDSLYPEDDL     453
```

Supplementary Figure S2

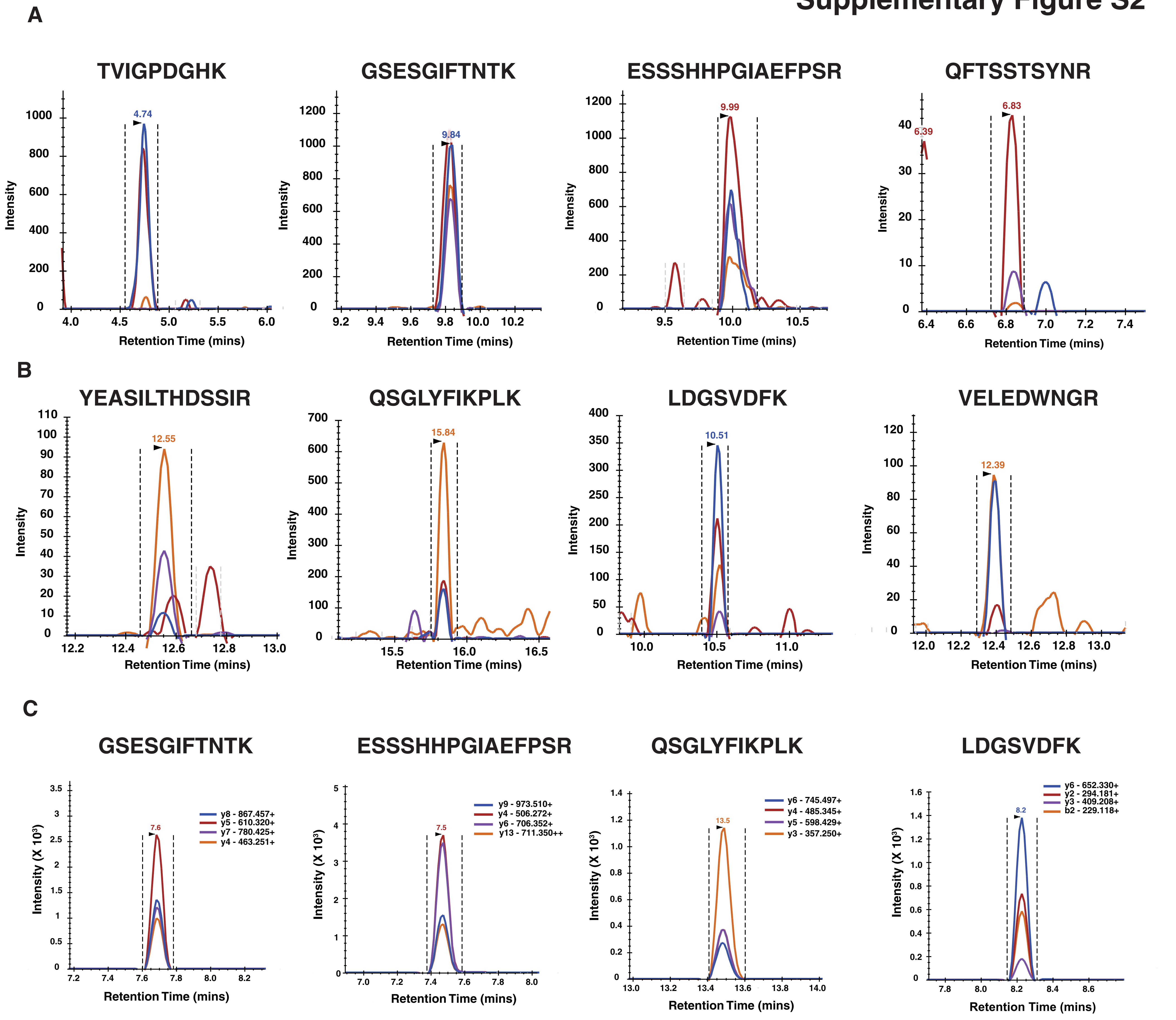

Supplementary Figure S3

A

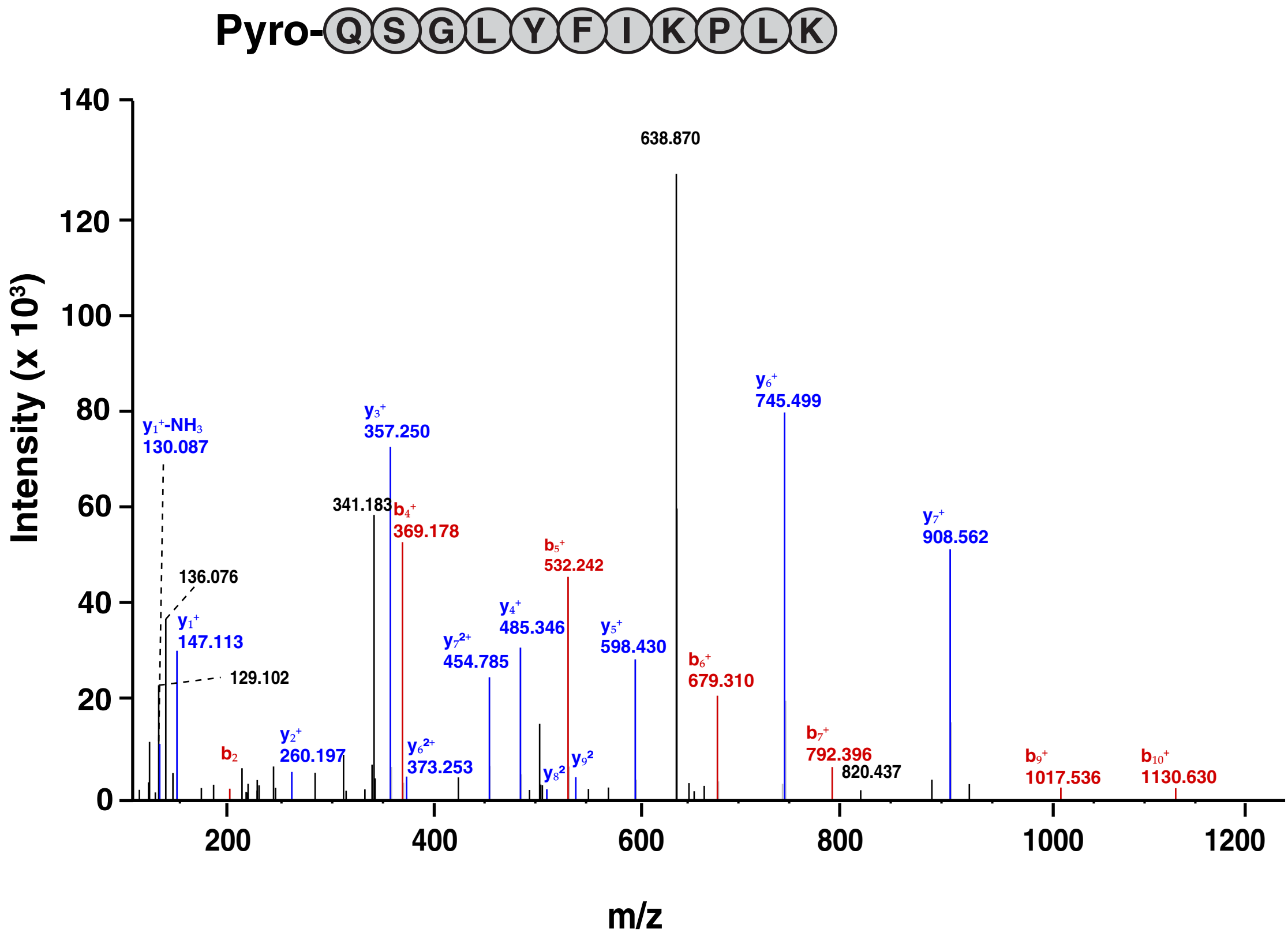

B

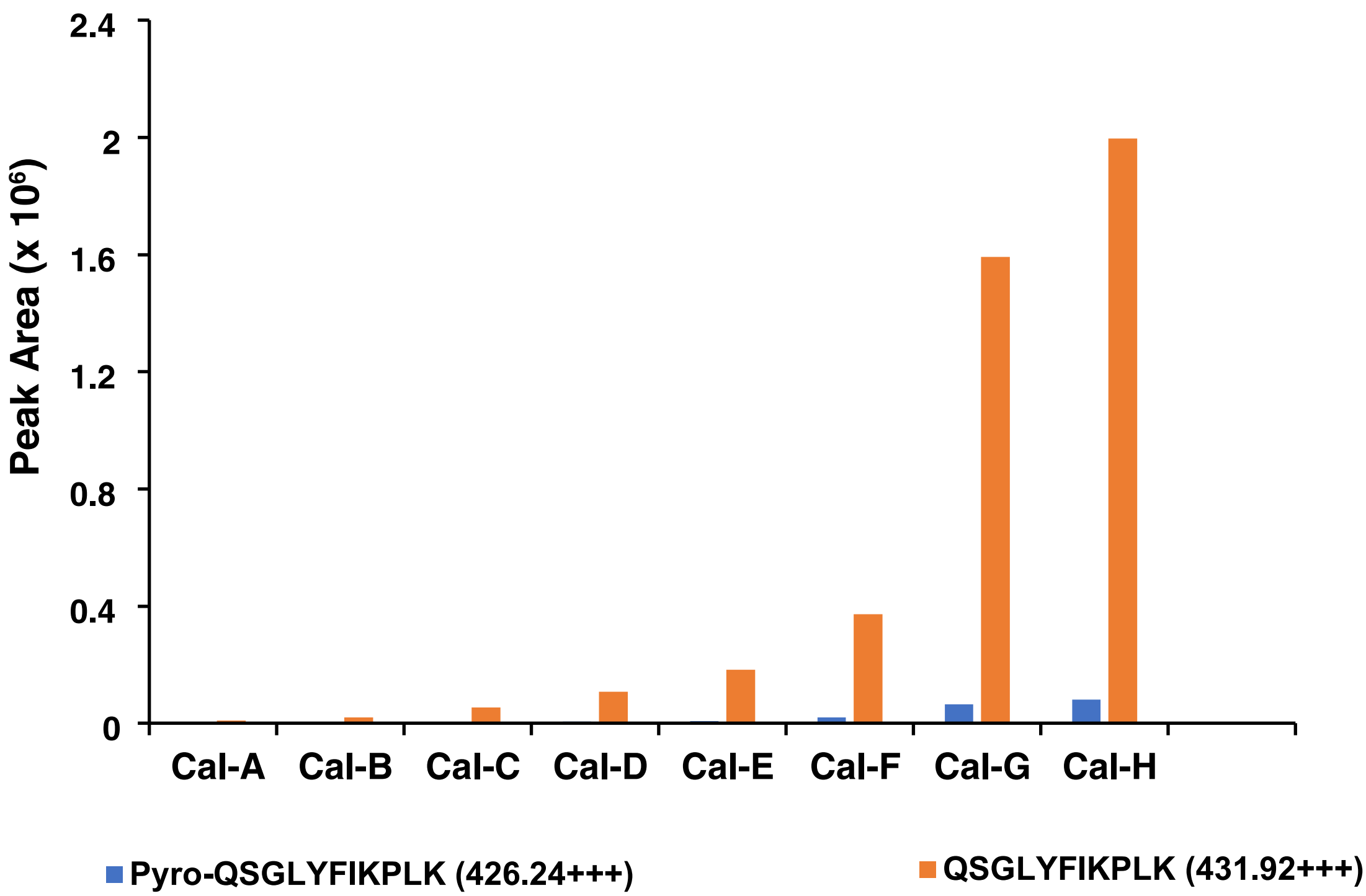

Supplementary Figure S4

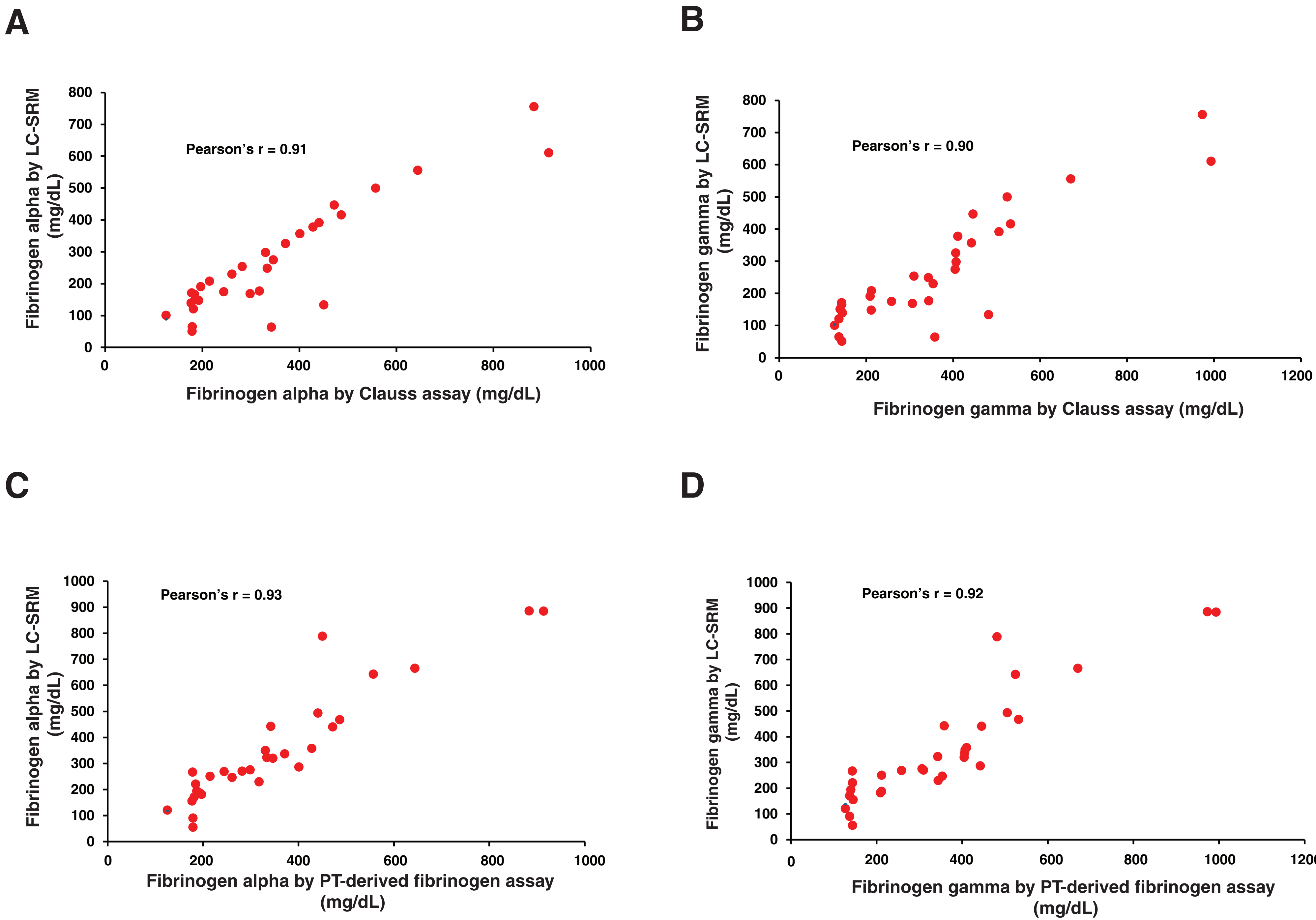

Supplement: Supplementary file 1 [file mmc1.pdf]
